# *Escherichia coli* RecG functionally suppresses human Bloom syndrome phenotypes

**DOI:** 10.1186/1471-2199-13-33

**Published:** 2012-10-30

**Authors:** Michael W Killen, Dawn M Stults, William A Wilson, Andrew J Pierce

**Affiliations:** 1 Department of Microbiology, Immunology and Molecular Genetics, Markey Cancer Center, University of Kentucky, Lexington, KY, USA; 2 Present address: Western Kentucky University, Bowling Green, KY, USA; 3 Sarah Cannon Research Institute, Nashville, TN, USA; 4 Department of Radiation Medicine, College of Medicine, University of Kentucky, Lexington, KY, USA; 5 Present address: MedImmune, LLC, Gaithersburg, MD, USA

## Abstract

Defects in the human *BLM* gene cause Bloom syndrome, notable for early development of tumors in a broad variety of tissues. On the basis of sequence similarity, *BLM* has been identified as one of the five human homologs of *RecQ* from *Escherichia coli*. Nevertheless, biochemical characterization of the BLM protein indicates far greater functional similarity to the *E. coli* RecG protein and there is no known RecG homolog in human cells. To explore the possibility that the shared biochemistries of BLM and RecG may represent an example of convergent evolution of cellular function where in humans BLM has evolved to fulfill the genomic stabilization role of RecG, we determined whether expression of RecG in human BLM-deficient cells could suppress established functional cellular Bloom syndrome phenotypes. We found that RecG can indeed largely suppress both the definitive elevated sister chromatid exchange phenotype and the more recently demonstrated gene cluster instability phenotype of BLM-deficient cells. In contrast, expression of RecG has no impact on either of these phenotypes in human cells with functional BLM protein. These results suggest that the combination of biochemical activities shared by RecG and BLM fill the same evolutionary niche in preserving genomic integrity without requiring exactly identical molecular mechanisms.

## Background

Human cells possess five proteins with clear sequence homology to the *E. coli* RecQ protein: BLM, WRN, RECQL, RECQL4 and RECQL5. These proteins are all implicated in preserving genomic integrity (reviewed in [1,2]). Functionally, inherited homozygous defects in *BLM*, *WRN* or *RECQL4* cause human disease: Bloom syndrome, Werner syndrome and Rothmund-Thomson/RAPADILINO/Baller-Gerold syndromes respectively. Bloom syndrome is particularly striking for its predisposition to early-onset malignancy with a broad distribution of cancer types similar to that seen with sporadic tumors in the general population [3].

Sequence homology of BLM with RecQ notwithstanding, characterization of the *in vitro* activities of BLM demonstrates significant similarities to the biochemistry of the *E. coli* RecG DNA translocase protein. Both BLM [4,5] and RecG [6,7] can bind to and regress multi-stranded DNA structures that model stalled replication forks. Similarly, both BLM [8,9] and RecG [10,11] have the capacity to bind to and branch migrate Holliday junctions. Both BLM [12,13] and RecG [14] have also been shown to dismantle D-loops where a 3^′^-OH ssDNA has invaded a homologous DNA duplex, although the mechanism by which RecG carries out this reaction is less well established [15]. The manner by which RecG accomplishes these tasks is in large part made clear by its crystal structure [16]: a RecG monomer binds at a model replication fork by inserting a C-terminal protein wedge domain into the fork. The body of RecG then functions as a double stranded DNA translocase to pull on and reanneal DNA template strands through the body of the protein. At such time as the nascent DNA strands encounter the wedge domain, they are stripped off and allowed to anneal together resulting in the formation of a Holliday junction. As RecG continues to translocate on the dsDNA, the branch point of the Holliday junction is effectively migrated. In the absence of high-resolution structural information it remains unclear precisely how BLM carries out these activities.

The BLM protein also possesses activities it is not known to share with RecG. BLM can act in concert with EXO1 at double stranded DNA ends to cause a 5^′^-3^′^ single stranded resection that exposes a free ssDNA 3^′^ end suitable for loading with Rad51 [17], reminiscent of the combined activities of the *E. coli* RecQ helicase and RecJ 5^′^-3^′^ exonuclease [18,19]. Alternatively, BLM can also functionally interact with the DNA2 exonuclease to carry out a similar reaction [20]. BLM has strong unwinding activity on G-quadruplex DNA structures [21] as well as both ssDNA annealing [22,23] and/or strand exchange activities [24]. Notably, BLM has many well-characterized protein-protein interactions, including those with RMI1, C16orf75 (RMI2) and TOP3A [25-28] that collectively mediate double-Holliday junction dissolution, as well as direct interaction with the Rad51 recombinase [29] and with the multi-component Fanconi anemia protein containing BRAFT complex [30]. In contrast, RecG functions in a largely monomeric manner [31].

The mechanistic similarities between BLM and RecG have led us and others [32] to speculate that *E. coli* RecG and human BLM may be functional analogs. In order to test this hypothesis and to determine the extent to which the shared biochemical activities of BLM and RecG are responsible for suppressing the functional cellular phenotypes observed in human cells lacking BLM, we reasoned that suitable expression of RecG might suppress a BLM defect. The best characterized cellular phenotype of BLM deficiency is a 10-fold elevated frequency of sister chromatid exchanges [33], thought to represent a hyper-recombination phenotype indicative of elevated crossing-over and overall genomic instability. In addition, we have recently demonstrated that BLM deficiency causes a striking destabilization of the highly repetitive human ribosomal RNA gene clusters (the ‘rDNA’), with recombination-mediated genomic restructuring of these clusters increased 100-fold over cells wild-type for BLM function [34]. Accordingly, we engineered several semi-humanized RecG protein expression systems and stably introduced these constructs into human cells either wild-type or defective for the BLM protein. We then assayed the resulting RecG transgene expressing cells for changes in these two phenotypes.

## Methods

### Protein expression constructs

**Figure 1:**
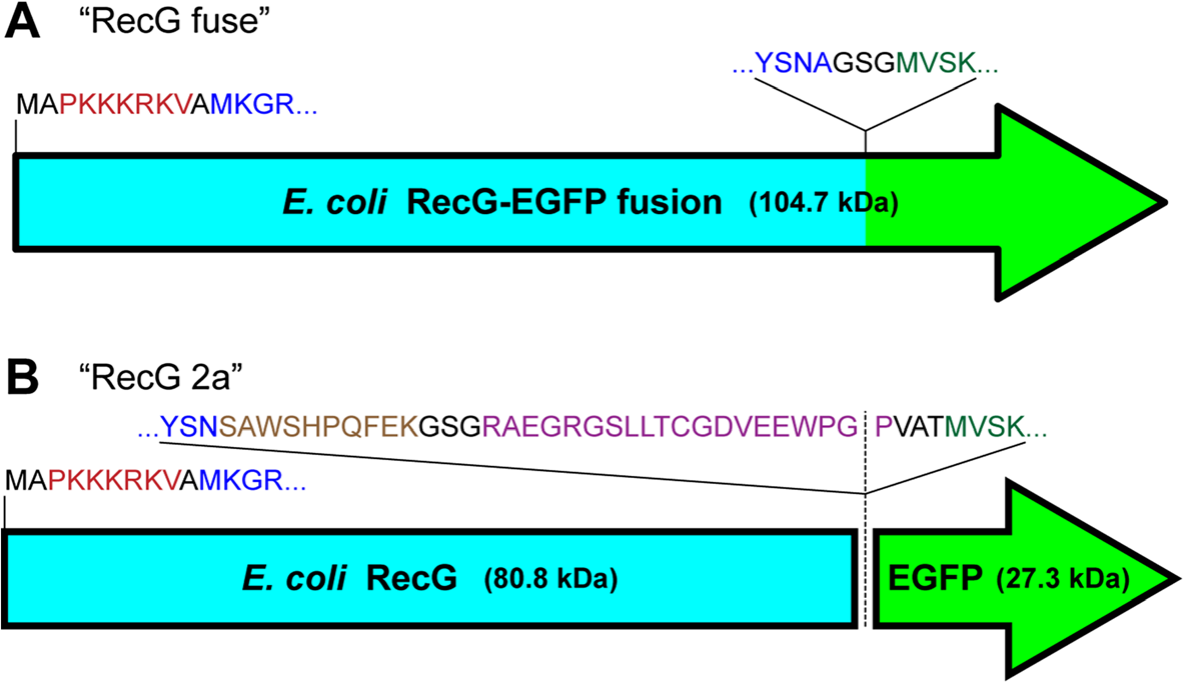
**RecG expression constructs.** 1**A**) RecG expressed as a fusion protein with EGFP. Lettering colors: black: linker amino acids, red: nuclear localization signal, blue: RecG coding sequence, green: EGFP coding sequence. 1**B**) RecG co-translated with EGFP as two separate polypeptides. Lettering colors: black: linker amino acids, red: nuclear localization signal, blue: RecG coding sequence, brown: strep-tag II, purple: 2a ‘self-cleaving’ peptide, green: EGFP coding sequence. Dotted line represents the division of the precursor polyprotein into two independent polypeptides. The full-length wild-type *E. coli* RecG polypeptide initiates with the indicated MKGR… peptide sequence and terminates with the indicated …YSNA peptide sequence.

The coding sequence for RecG was isolated from *E. coli* TOP10 (Invitrogen) genomic DNA with the addition of a consensus Kozak sequence [35] and the SV40 large T-antigen nuclear localization signal (PKKKRKV) by PCR using primers 5^′^-ggggggggatccagccaccatggctccaaaaaaaaagcgcaaagtggcgatgaaaggtcgcctg and 5^′^-gggggggatatcgcggccgcttacgcattcgagta (RecG sequences underlined) followed by cloning into the pCAGGS mammalian constitutive expression vector. A cryptic polyadenylation sequence in the RecG coding sequence 5^′^-AATAAA (AsnLys) was removed via silent mutagenesis to 5^′^-AACAAG (AsnLys). Carboxy-terminal additions of the enhanced green fluorescent protein (EGFP) coding sequence were constructed either to produce a RecG-EGFP fusion protein with a GSG linker peptide (Figure 1A) “RecG fuse” (predicted molecular weight: 104.7 kDa), or to produce two proteins in a bi-cistronic manner: RecG fused to the Strep-tag II for potential affinity purification [36] followed by a GSG linker, the 2A polyprotein ‘self-cleaving’ sequence from *Thosea asigna* virus [37,38], a VAT peptide linker and EGFP (Figure 1B) “RecG 2a”. In the bi-cistronic construct the predicted molecular weight of the RecG polypeptide is 80.8 kDa and the predicted molecular weight of the EGFP polypeptide is 27.3 kDa. All final construct sequences were verified by direct DNA sequencing. The finished constructs express essentially the entirety of the wild-type *E. coli* RecG polypeptide, with small amino- and carboxy-terminal extensions. The EGFP expression vector used for subcloning the EGFP coding sequence was pEGFP-N1 (Clontech). The RecG expression constructs with full annotation are available from the non-profit Addgene plasmid repository ( http://www.addgene.org) as plasmids #31274 and #31275.

### Cell lines

Cell lines used that express normal BLM include the SV40-transformed fibroblast line GM00637 (Coriell Cell Repositories) “*BLM+*” and the cervical carcinoma line HeLa S3 (ATCC: American Type Culture Collection) “HeLa”. Cell lines used that are deficient in BLM protein include GM08505 (Coriell Cell Repositories) which are SV40 transformed fibroblasts derived from a Bloom syndrome patient homozygous for the Ashkenazi Jewish founder BLM mutation (6-bp del/7-bp ins) at nucleotide 2281 of the open reading frame, hereafter referred to as “*BLM-*”. A second BLM deficient line used was GM16375 (Coriell Cell Repositories) which are EBV transformed lymphocytes from a French-Canadian Bloom syndrome patient homozygous for a C>A transversion resulting in a (S595X) termination mutation, hereafter referred to as “*BLM- *FC”. The BLM-defective line stably suppressed by either BLM cDNA expression, or by a control empty vector are the lines PSNF5 “*BLM-*: cDNA” and PSNG13 “*BLM-*: empty vector” respectively, as described in [39] (kind gift from Ian Hickson) both derived originally from the GM08505 line (Coriell Cell Repositories). Lines were generally grown in MEM with 10% fetal bovine serum, with *L-gln* and antibiotic supplementation at 37C in a humidified 5% CO_2_ incubator.

Stable cell lines were generated by electroporating either a RecG expression construct or a control EGFP expression construct into cells, followed by unselected cell population expansion, one round of flow-sorting enrichment for green fluorescent cells, further unselected expansion, and finally a second round of flow-sorting enrichment for green fluorescent cells. Clonal and subclonal derivatives of these highly enriched fluorescent populations were subsequently derived by limiting dilution. All of the transgene expressing cell lines generated and used in this work are either clonal or subclonal, with the exception of the “*BLM-* FC: RecG fuse” line: *BLM-* FC cells were transduced by a high-titer lentivirus (Welgen, Inc.) containing an expression cassette for the RecG-EGFP fusion construct (Figure 1A) and separated into green fluorescent and non-fluorescent populations by flow sorting, and the *BLM-*: EGFP line which was isolated on the basis of drug resistance only. Stable cell lines were chosen for further experiments on the basis of the levels of EGFP expressed in these lines, as measured by flow cytometry.

### Sister chromatid exchange assays

Sister chromatid exchange assays were performed largely according to [40] with minor modifications [34]. Cellular metaphase spreads were imaged and scored individually by counting the number of visible exchanges and the number of chromosomes in each unique high powered microscope field to calculate the number of SCEs per chromosome. In our hands, scoring the number of SCEs per chromosome is more robust than scoring the number of SCEs per metaphase and is relatively insensitive to the ploidy of the cell line under investigation [41]. The resulting SCEs/chromosome figures were binned and plotted. All statistical tests were performed using unbinned data. SCE experiments were generally performed by collecting fixed metaphase cells from one or two experiments, which were subsequently dropped on microscope slides to release the metaphase spreads, stained and counted together in a single session at the microscope.

### Western blotting

Protein extracts were prepared using RIPA buffer as described previously [34]. All resolving SDS-PAGE gels used 9% acrylamide and were blotted onto Hybond-ECL nitrocellulose membrane (Amersham Biosciences, cat. #RPN68D). Primary antibodies used were: rabbit anti-GFP (Cell Signaling Technologies, cat. #2555), rabbit anti-beta-actin (Cell Signaling Technologies, cat. #4970), rabbit anti-beta-tubulin (NeoMarkers, cat. #RB-9249-PO). The secondary antibody was ImmunoPure Antibody donkey anti-rabbit IgG conjugated with horseradish peroxidase (Pierce, cat. #31458). Blots were developed using an Amersham™ ECL Plus western blotting detection system (GE Healthcare, cat. #RPN2132) and imaged with a Storm 860 PhosphorImager (Molecular Dynamics).

### GCI analysis

Gene cluster instability analysis was carried out as described previously [34]. Briefly, genomic DNA was prepared in the solid phase by digesting single cell suspensions in agarose with proteinase K in the presence of sarkosyl and EDTA, rinsed thoroughly and equilibrated in 50% glycerol/10 mM Tris/1 mM EDTA pH 8.0 and stored at -20C. 10 μl agarose slices containing approximately 1 μg genomic DNA were equilibrated in suitable restriction digestion buffer and digested overnight with EcoRV (New England Biolabs). Digested DNA still in solid form was loaded into a 1% PFC agarose (Bio-Rad) gel and run in 0.5× TBE buffer (44.5 mM Tris base, 44.5 mM boric acid, 1 mM EDTA pH 8.0) using a CHEF-MAPPER system (Bio-Rad) at 14C. Pulsed-field electrophoretic conditions were a field strength of 6 V/cm with 120° separation between field vectors. Field switch times varied from 3 seconds to 90 seconds with a ‘ramp factor’ of 0.357. Gels were run for 24 hours, equilibrated to 0.5% glycerol in water, then dried, rehydrated, probed with a radiolabeled probe specific for the human rDNA [42] and imaged with a Storm 860 PhosphorImager (Molecular Dynamics).

### EGFP quantitation by flow cytometry

For cell lines carrying EGFP transgenes, the fold increase in fluorescence relative to non-fluorescent cells was calculated by dividing the geometric mean value of green fluorescence emitted from the fluorescent cell sub-population by the geometric mean value of background green autofluorescence from the non-fluorescent sub-population of the same cell line.

## Results

### RecG expression suppresses the *BLM-* elevated SCE phenotype

**Figure 2:**
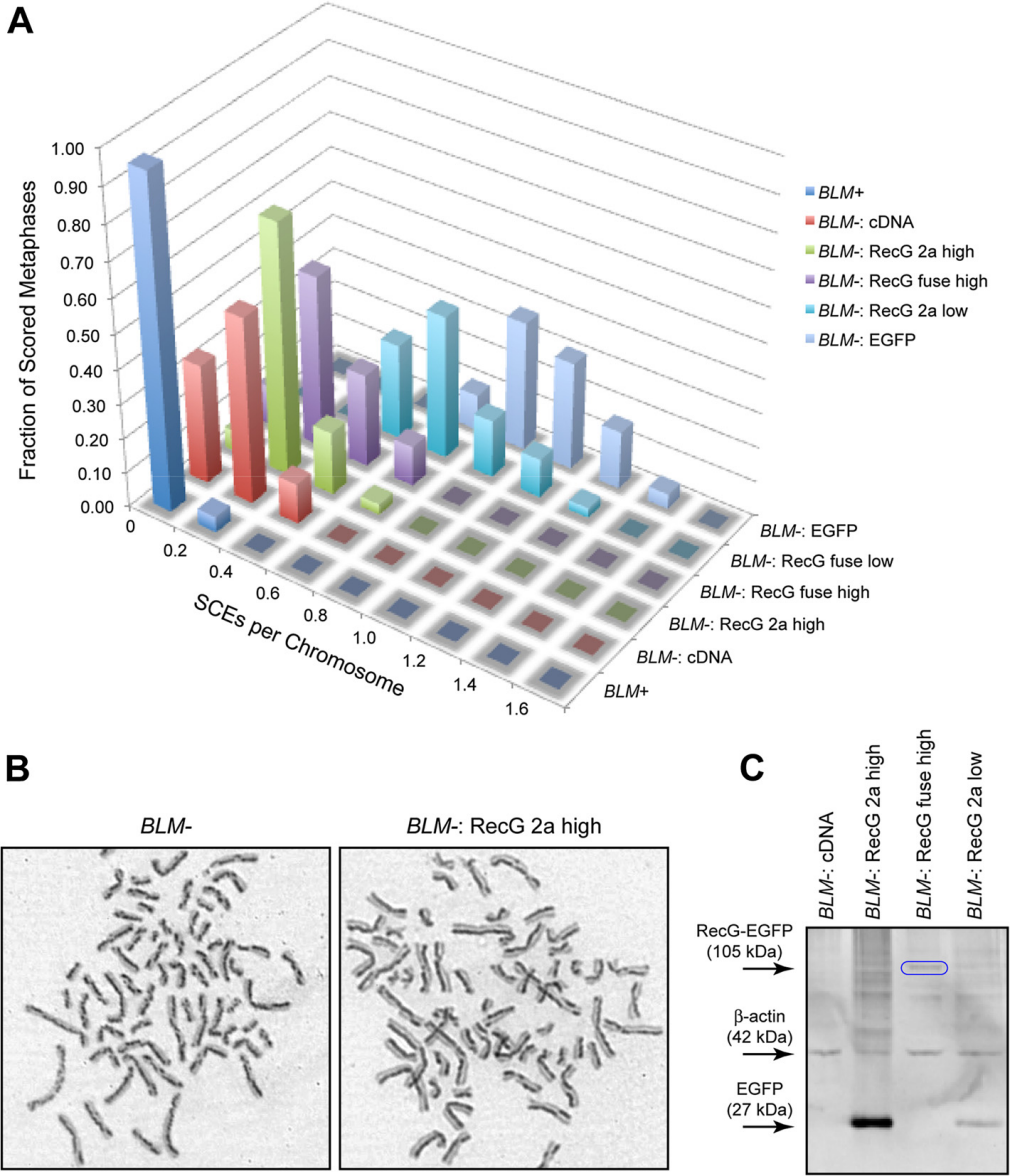
**RecG expression suppresses the *BLM-*elevated SCE phenotype.** 2**A**) Normalized sister chromatid exchange frequencies. “RecG 2a” denotes cells expressing RecG and EGFP as separate co-translated proteins. “RecG fuse” denotes cells expressing RecG and EGFP together as a fusion protein. 2**B**) Representative sister chromatid exchange metaphase spreads with sister chromatids differentially stained. The harlequin staining pattern of the *BLM-* cells is greatly reduced upon expression of RecG. 2**C**) RecG expression levels. Protein extracts from the indicated cell lines are blotted with an anti-GFP antibody and with anti-β-actin as a loading control. RecG-EGFP fusion proteins (105 kDa) are indicated with blue ovals. EGFP is detected as a proxy biomarker for RecG in the “RecG 2a” co-translated lines. See also Table 1 for EGFP quantitation by flow cytometry.

**Figure 3:**
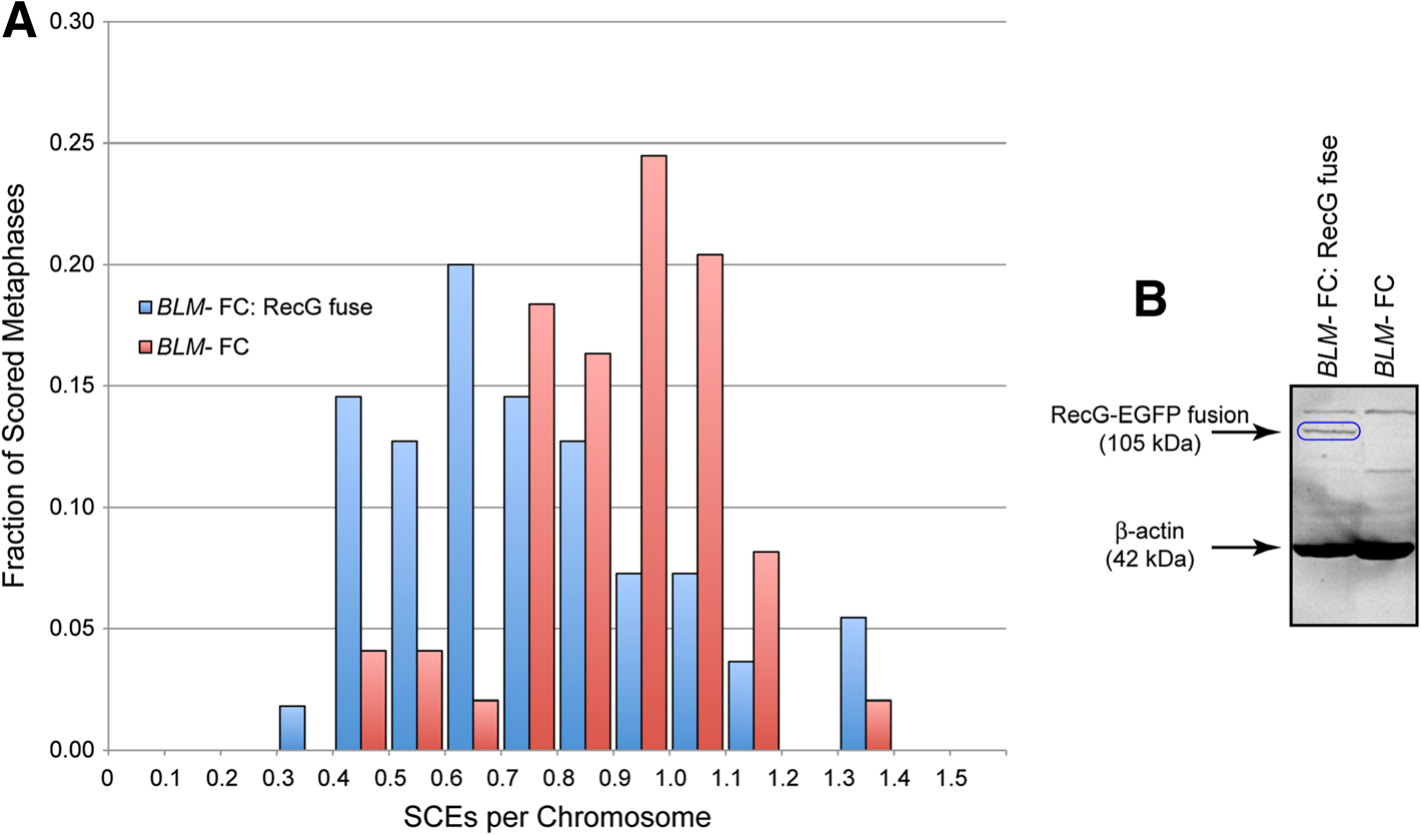
**RecG expression reduces SCE in a French-Canadian *BLM-*line.** 3**A**: Normalized sister chromatid exchange frequencies. “*BLM-* FC”: EBV-immortalized lymphocytes homozygous for an inactivating (S595X) mutation. “*BLM-* FC: RecG fuse”: the same cells expressing low levels of the RecG-EGFP fusion protein. 3**B**: Western blot showing relative protein levels. Blue oval: RecG-EGFP fusion protein. “*BLM-* FC: RecG fuse”: protein extract from French-Canadian *BLM-* cells stably expressing low levels of the RecG-EGFP fusion protein. “*BLM-* FC”: protein from French-Canadian *BLM-* cells lacking a RecG transgene. See also Table 1 for EGFP quantitation by flow cytometry.

We stably transfected *E. coli* RecG expressing transgenes into human cells with both BLM-defective and BLM-normal genetic backgrounds. Two different expression systems were used, a direct fusion protein between RecG and EGFP, and a bi-cistronic system where separate RecG and EGFP polypeptides are co-translated. RecG fusion proteins in *E. coli* largely retain functionality [43], and indeed the RecG-EGFP fusion protein plasmid used in this work increases the resistance of deletion recG *E. coli* to mitomycin C (Robert Lloyd, personal communication). We found a significant RecG dose-dependent decrease in SCEs per chromosome in *BLM-* cells (Table 1, Figure 2**)** with the highest level of RecG expression tested reducing the elevation of SCEs by 75%. In our hands, expression of the human BLM protein from a cDNA construct in *BLM-* cells reduces this characteristic elevated SCE phenotype by 80% (Figure 2A, Table 1 and [34]), so *E. coli* RecG expression is nearly as effective as the human protein in this system. RecG suppressed the elevated *BLM-* SCE phenotype either when expressed as an EGFP fusion protein or as a separate polypeptide co-translated with EGFP. In all cases where RecG is expressed in a *BLM*- line, mean SCEs/chromosome are reduced with high confidence (two-tailed Mann–Whitney test, P<0.001). Expression of EGFP alone without RecG in *BLM-* cells has no statistically significant effect on SCE levels (Figure 2A, Table 1). That RecG suppression of the *BLM-* elevated SCE phenotype is not dependent on the genetic background of the cells other than with respect to the loss of BLM is demonstrated by manner in which RecG suppresses sister chromatid phenotypes both in cells of Ashkenazi Jewish origin and separately in cells of French-Canadian origin (Figure 3A). Both of these lines are essentially BLM-null due to a 6-bp deletion/7-bp insertion, and due to an S595X mutation respectively yet presumably have otherwise heterogeneous genetic backgrounds. RecG is expressed at a low level in “*BLM-* FC: RecG fuse” cells (Figure 3B, Table 1), resulting in a less dramatic, 25% reduction in the elevated SCE phenotype, nevertheless in a statistically highly significant manner (P=0.0004, Mann–Whitney two-tailed test).

**Table 1:** SCE levels and EGFP expression

**Cells**	**SCE/Chr**	**EGFP**	**% EGFP+**	**HPF**	**Xomes**
HeLa	0.09	1.0	0	21	605
HeLa: RecG fuse	0.08	42.6	98	32	835
*BLM+*	0.10	1.0	0	22	863
*BLM+*: RecG 2a	0.10	49.3	100	30	999
*BLM-*: cDNA	0.28	1.0	0	26	1625
*BLM-*: RecG 2a high	0.33	148	93	33	1861
*BLM-*: RecG fuse high	0.34	29.8	87	26	1149
*BLM-*: RecG 2a low	0.68	12.4	92	36	2017
*BLM-*: EGFP	1.02	27.3	57	48	3013
*BLM-*: empty vector	1.05	1.0	0	25	1354
*BLM-*	0.98	1.0	0	16	883
*BLM-* FC: RecG fuse	0.70	6.4	100	55	1981
*BLM-* FC	0.91	1.0	0	49	2077
*BLM-*: RecG fuse med	0.43	30.6	87	29	1925
*BLM-*: lost RecG fuse	0.86	8.96	1	25	1316

Cells: The stable cell line being investigated.

SCE/Chr: Median number of sister chromatid exchanges observed per chromosome (sister chromatid pair).

EGFP: Relative levels of fluorescence for cells expressing RecG and/or EGFP, relative to cells not expressing EGFP.

% EGFP+: The fraction of cells in a population with above-background green fluorescence due to expression of EGFP.

HPF: The number of high powered microscope fields scored.

Xomes: The total number of sister chromatid pairs scored for exchanges.

### RecG has no effect on SCE levels in cells expressing normal BLM protein

**Figure 4:**
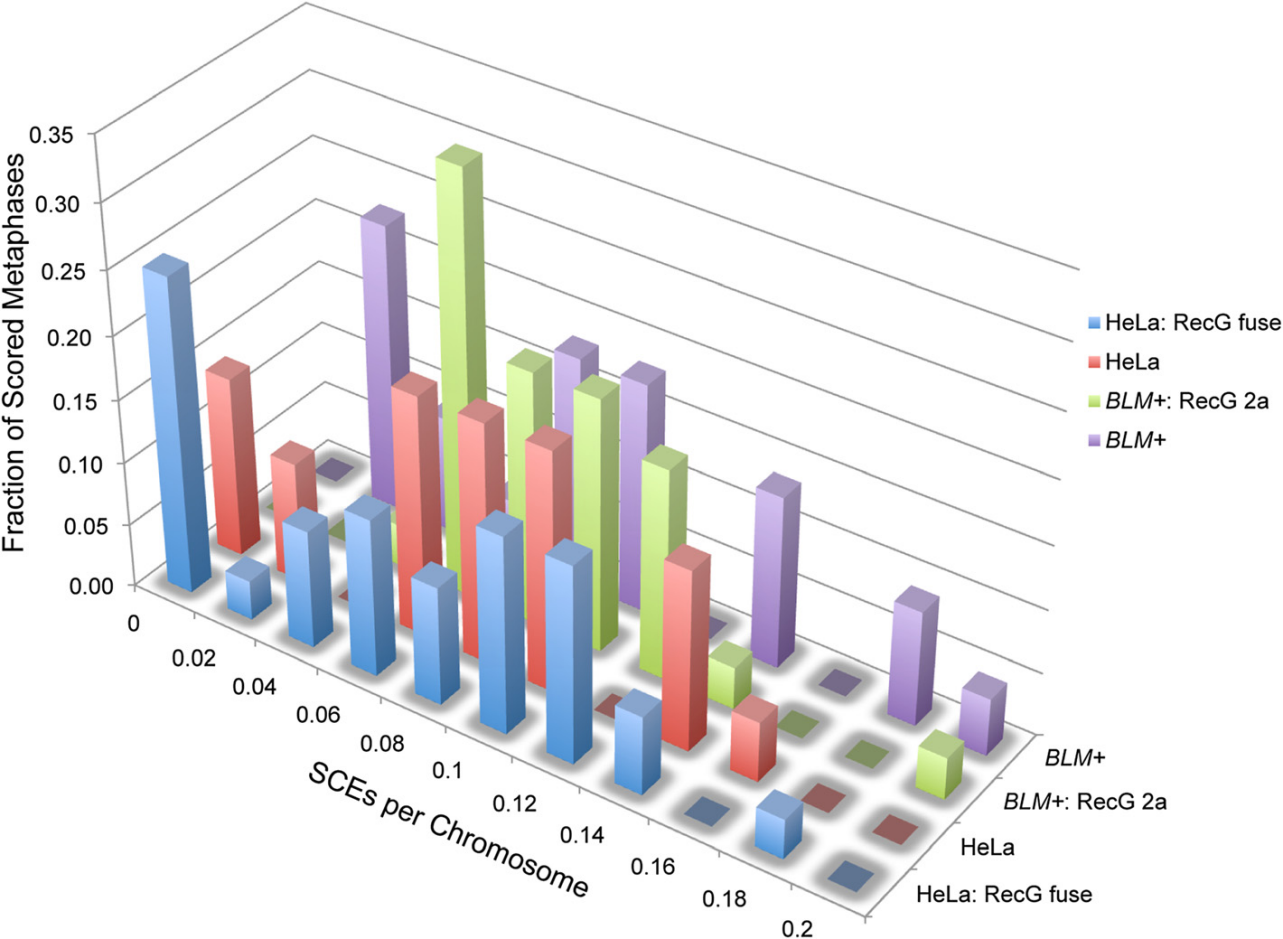
**RecG expression has no effect on SCE levels in cells with normal BLM protein.** Normalized sister chromatid exchange frequencies are shown. Note the scale of the x-axis in this figure ranges from 0 to 0.2 for the distribution of SCEs/chromosome in lines with normal BLM function, whereas the scale of the x-axis in Figure 2A ranges from 0 to 2.0 SCEs/chromosome, consistent with the known 10-fold elevation of sister chromatid exchanges in *BLM-* lines.

In order to establish the *BLM-* specificity of the RecG SCE reduction effect, we carefully examined SCE levels in two different *BLM+* lines that stably express relatively high levels of either the RecG-EGFP fusion protein, or the bicistronic RecG/EGFP construct (Figure 4). We found that RecG expression had no statistically significant effect on either the median levels of SCEs per chromosome or in the distribution of SCEs seen in either *BLM+* line (Table 1).

### Loss of RecG expression restores the elevated SCE phenotype to *BLM-* cells

**Figure 5:**
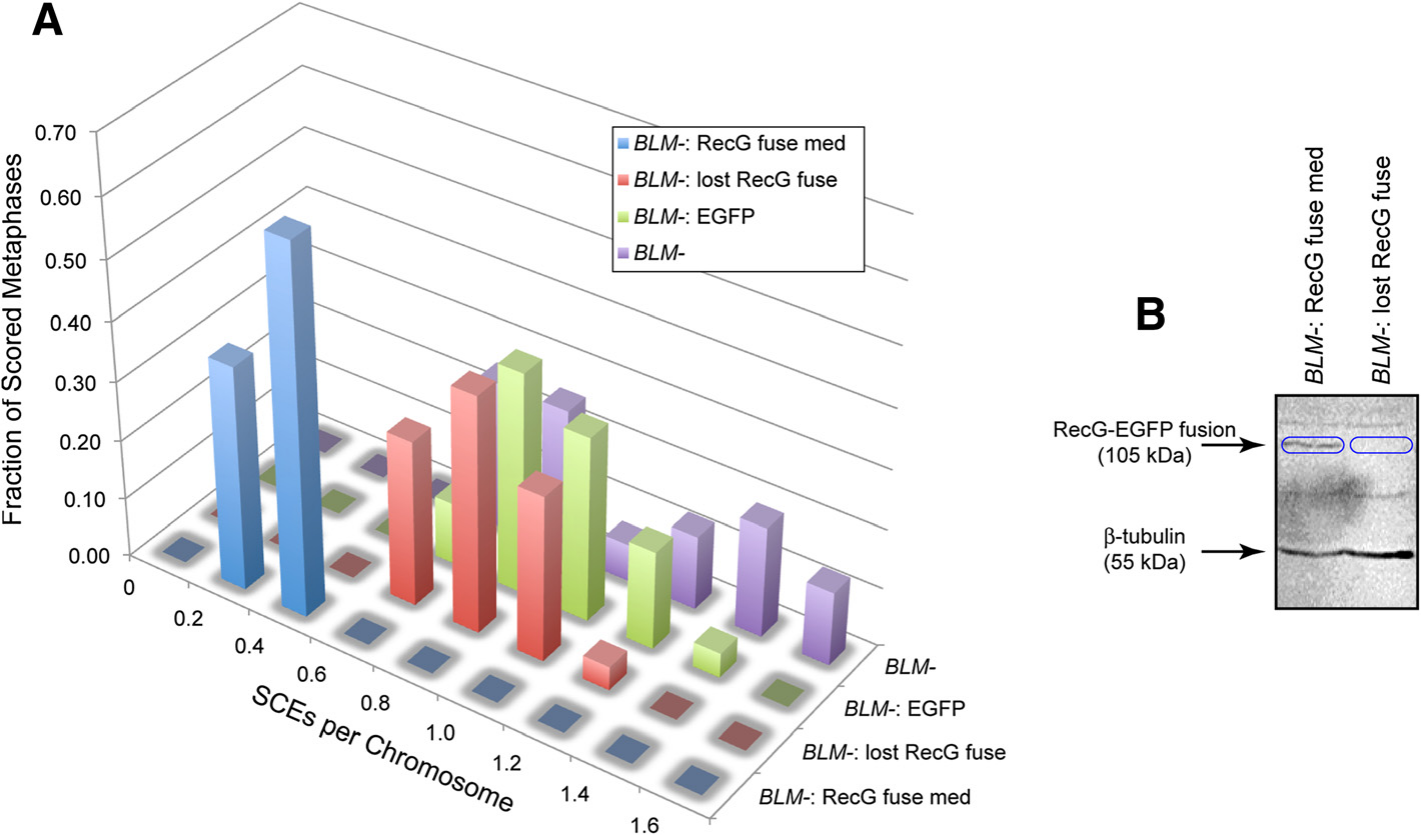
**Loss of RecG expression in *BLM-*cells restores the elevated SCE phenotype.** 5**A**) Normalized sister chromatid exchange frequencies are shown. 5**B**) Loss of expression of RecG-fuse is shown by western blotting (blue ovals). See also Table 1 for EGFP quantitation by flow cytometry.

One of the clonal *BLM-* lines stably expressing medium levels of the RecG-EGFP fusion protein (Figure 5B, “*BLM-*: RecG fuse med”) gradually lost EGFP expression during two months of continuous cell culture in the absence of selection, likely due to epigenetic silencing of the RecG-EGFP transgene. In the resulting “*BLM-*: lost RecG fuse” line, SCE levels were re-elevated to nearly those of either the parental *BLM-* line, or the control *BLM-* line containing a transgene for EGFP alone “*BLM-*: EGFP” (Figure 5A, Table 1), confirming that RecG expression is solely responsible for suppressing the elevated SCE phenotype in *BLM-* cells.

### RecG expression reduces the elevated gene cluster instability (GCI) of *BLM-* cells

**Figure 6:**
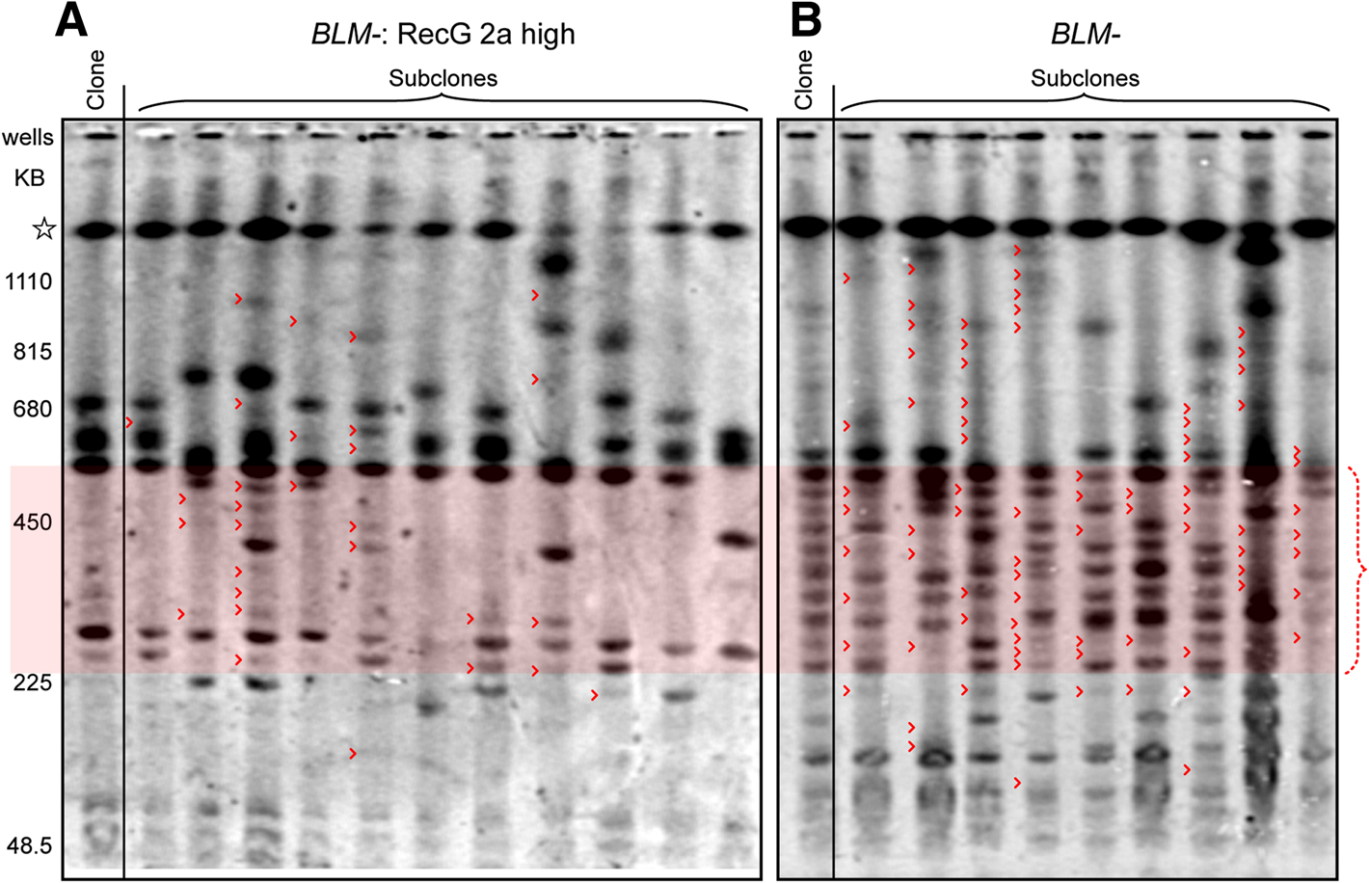
**RecG stabilizes the gene cluster instability of *BLM-*cells.** Southern blotting detection of the rDNA clusters with resolution up to 1 Mb is shown. Cluster lengths are calibrated by *S. cerevisiae* chromosome size markers. Open star is the 1 Mb resolution limit of the gel: all clusters larger than 1 Mb co-localize to this band. Clonal populations are shown on the left of each panel, with a collection of subclones derived from this clonal population to the right. Red carets: mitotic recombination indicated by minor intensity gene cluster bands. The zone of particularly high instability in the 250 kb to 550 kb size range is indicated by red shading and by the red dotted bracket at the right. 6**A**) “*BLM-*: RecG 2a high”: *BLM-* cells stably expressing higher levels of cotranslated RecG/EGFP. 6**B**) The parental *BLM-* line from which the “*BLM-*: RecG 2a high” cells were derived.

Loss of BLM very strongly increases the rate of spontaneous recombination-mediated genomic restructuring in the highly repetitive human rRNA gene clusters [34]. In *BLM-* cells, recombination alters gene cluster lengths on the order of every round of mitotic cell division, an approximate 100-fold increase over the rate in *BLM+* cells. This rapid randomization of cluster lengths produces a diagnostic ladder-like electrophoretic karyotype with each rung on the ladder separated by the 48.5 kb unit length of the human rDNA repeat, particularly in the range of gene cluster lengths from 250 kb to 550 kb (Figure 6 – shaded in red, red dotted side bracket). The intensity of individual cluster bands is related to how early in the clonal expansion the mitotic recombination event took place, with more intense bands having arisen earlier in the expansion of a clonal population [34]. Expression of RecG suppresses this elevated GCI phenotype: the rate of spontaneous cluster restructuring is greatly reduced (Figure 6 – red carets) although as with suppression of the elevated SCE phenotype of *BLM-* cells, there is a residual low level of GCI even at the highest levels of RecG expression assayed. Notably, expression of RecG is particularly effective at restabilizing gene clusters in the previously highly unstable 250 kb to 550 kb size range, as seen by the significant reduction of gene cluster lengths detected in this range. When GCI is quantified by the average number of minor bands observed per clone [34], expression of RecG suppresses the elevated BLM GCI phenotype by more than 3-fold overall, from 8.7 minor bands per clone in the *BLM-* cells to 2.6 minor bands per clone in the *BLM-* cells that express RecG.

### RecG expression does not affect gene cluster instability in *BLM+* cells

**Figure 7:**
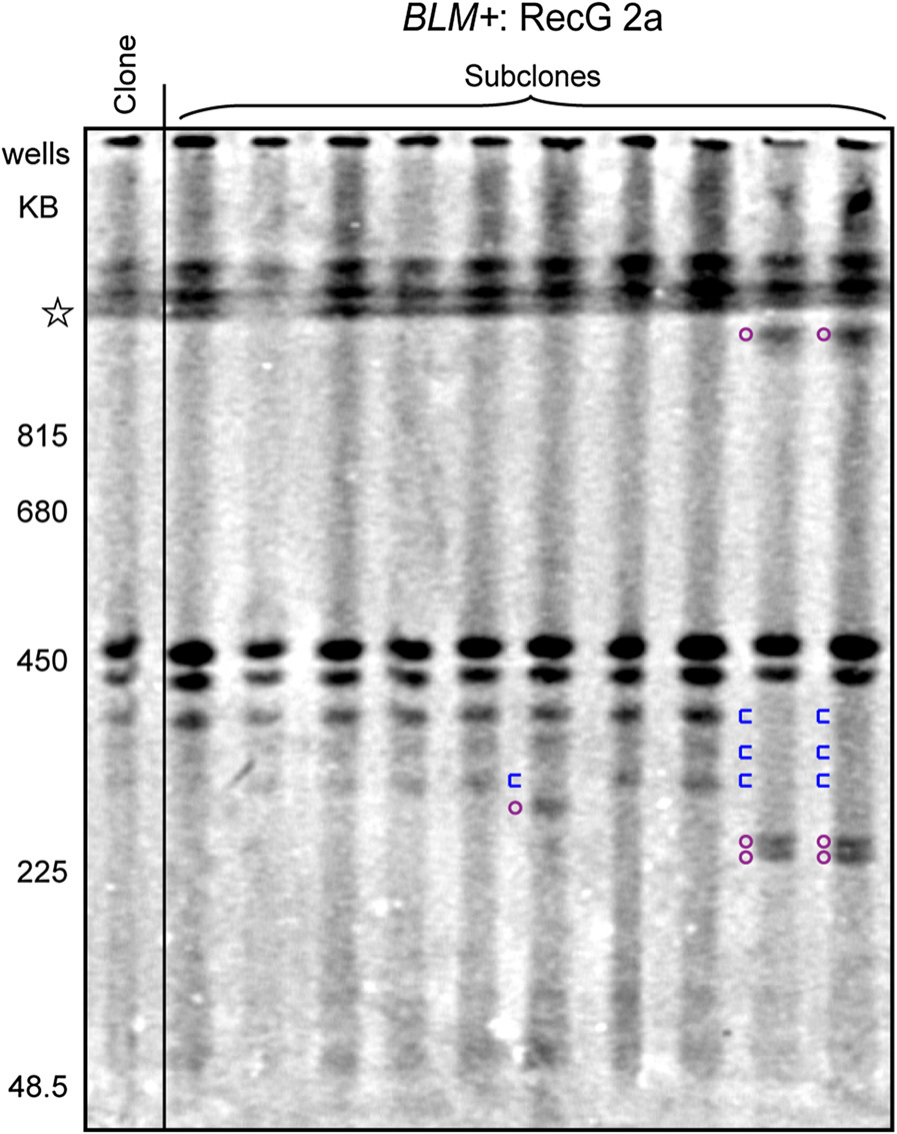
**RecG has no effect on gene cluster instability in cells with normal BLM.** Southern blotting detection of the rDNA clusters in *BLM+* cells stably expressing higher levels of co-translated RecG/EGFP, “*BLM+*: RecG 2a”. Cluster lengths resolved up to 1 Mb are shown. Cluster lengths are calibrated by *S. cerevisiae* chromosome size markers. Open star is the 1 Mb resolution limit of the gel: all clusters larger than 1 Mb co-localize in this region. A clonal cell population is shown in the far left lane of the gel. A collection of subclonal populations each derived from a single cell of this clonal population are shown to the right. Plum circles: relocated major band clusters. Blue braces: former location of major band clusters from the parental clone.

Expression of RecG as an independent polypeptide co-translated with EGFP did not have an obvious effect on gene cluster instability in a cell line wild-type for BLM protein (Figure 7) even though RecG is expressed at a relatively high level when measured by flow cytometry detection of the co-translated EGFP protein in this line (Table 1: *BLM*+: RecG 2a). The changes to the banding pattern in the subclones relative to that of the parental clonal population represent mitotic recombination events that occurred sufficiently late in the expansion of the parental clonal population as to be undetectable as visible minor-intensity banding. The relatively small number of changes to major intensity bands in the subclones and the lack of detectable minor bands in any of the cell populations is consistent with the low levels of spontaneous gene cluster restructuring seen in other *BLM+* transformed human cell lines [34].

## Discussion

**Figure 8:**
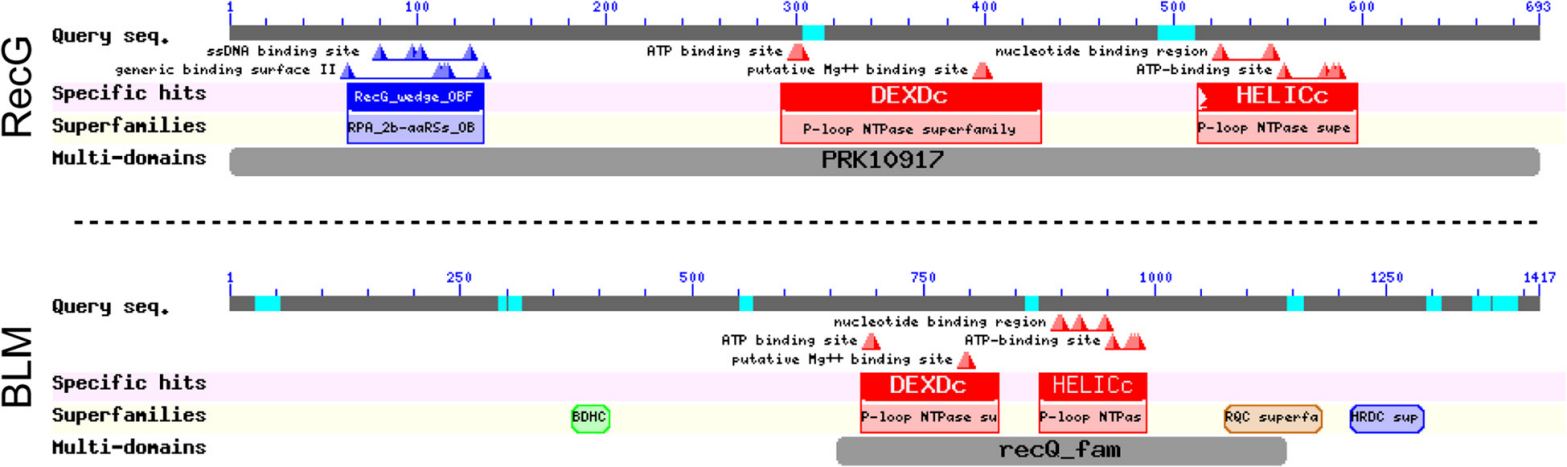
**Domain-based alignment of *E. coli*RecG and human BLM proteins.** All conserved domain analysis below is from [44], RecG accession GI:42669, BLM accession GI:4557365. RecG Conserved Domains: RecG_wedge_OBF: A subfamily of OB folds corresponding to the OB fold found in the N-terminal (wedge) domain of Escherichia coli RecG. DEXDc: DEAD-like helicases superfamily. A diverse family of proteins involved in ATP-dependent RNA or DNA unwinding. This domain contains the ATP-binding region. HELICc: Helicase superfamily c-terminal domain; associated with DEXDc-, DEAD-, and DEAH-box proteins, yeast initiation factor 4A, Ski2p, and Hepatitis C virus NS3 helicases. PRK10917: ATP-dependent DNA helicase RecG; Provisional BLM Conserved Domains: BDHCT: This is a C-terminal domain in Bloom’s syndrome DEAD helicase subfamily. DEXDc: DEAD-like helicases superfamily. A diverse family of proteins involved in ATP-dependent RNA or DNA unwinding. This domain contains the ATP-binding region. HELICc: Helicase superfamily c-terminal domain; associated with DEXDc-, DEAD-, and DEAH-box proteins, yeast initiation factor 4A, Ski2p, and Hepatitis C virus NS3 helicases. RQC domain: This DNA-binding domain is found in the RecQ helicase among others and has a helix-turn-helix structure. The RQC domain, found only in RecQ family enzymes, is a high affinity G4 DNA binding domain. HRDC domain: The HRDC (Helicase and RNase D C-terminal) domain has a putative role in nucleic acid binding. recQ_fam: ATP-dependent DNA helicase, RecQ family. All proteins in this family for which functions are known are 3^′^-5^′^ DNA-DNA helicases. These proteins are used for recombination, recombinational repair, and possibly maintenance of chromosome stability. This family is based on the phylogenomic analysis of JA Eisen (1999, Ph.D. Thesis, Stanford University).

We show here that expression of the *E. coli* RecG protein is able to largely, but not completely, suppress two functional human cellular phenotypes of BLM deficiency: elevated sister chromatid exchange, and elevated gene cluster instability. Although this result is in accord with the several shared biochemical activities of these two proteins, this functional suppression across different kingdoms of biology is interesting, particularly when considering that there appears to be no relationship of evolutionary sequence homology between RecG and BLM. Both RecG and BLM are members of the DEXDc superfamily of helicase proteins, and both contain both DEXDc and HELICc subdomains (Figure 8 and [44]), however, the *E. coli* RecQ protein is the clear evolutionary homolog of BLM on the basis of sequence similarity within these two conserved domains. The helicase family membership of RecG notwithstanding, RecG does not primarily fulfill the classical function of a helicase but rather seems to have co-opted the molecular engine of a helicase to translocate on double stranded DNA. We suggest that this manner of translocation may also be true of BLM particularly during the branch migration of Holliday junctions since the crystal structure of a Holliday junction shows minimal single-stranded DNA character [45]. Both RecG and BLM have extended amino acid sequences N-terminal to their DEXDc superfamily domains and it is possible that significant structure/function similarities to RecG may reside in this region. Alternatively, BLM may maintain a portion of the Holliday junction in transient single-stranded form as Holliday junctions branch migrate together during junction dissolution.

The *E. coli* RecG protein, with essentially no sequence similarity to human BLM, would seem unlikely to engage in any of the well-characterized protein-protein interactions that are important for function of the BLM protein in the wild-type human cellular context, although we have not experimentally ruled out these interactions. The capacity with which RecG can suppress *BLM*- cellular phenotypes suggests therefore that the physiological role of BLM in suppressing the two phenotype we investigated, sister chromatid exchanges and gene cluster instability, is to perform the same molecular reactions that can be performed in a human cell by the RecG monomer alone, namely direct manipulations of DNA structures. This is not to rule out a role for BLM and associated protein-protein interactions in suppression of other manifestations of genomic instability, or potentially BLM accessory proteins may help localize BLM to DNA structures upon which both BLM and RecG can act, thereby reducing the required level of BLM in the cell relative to the large amount of ectopic RecG expressed in the cell lines of this study. Activities of BLM not known to be shared by RecG, such as the unwinding of G-quadruplex DNA, must play a minor role in both sister chromatid exchange and gene cluster instability suppression assayed herein, or alternatively, a G-quadruplex activity of RecG may remain to be discovered.

We suggest that RecG and BLM fulfill the same physiological function in *E. coli* and human cells respectively: they recognize stalled/regressed DNA replication forks and then utilize their common biochemical functions to mitigate this topological genomic damage. Given the well-established biochemical similarities between the two proteins and the capacity for RecG to functionally substitute for BLM shown in this work, we consider this the most parsimonious conclusion. Nevertheless, an alternative possibility consistent with our data is that the lesions recognized by BLM and RecG may be distinct from each other, but that may be converted though subsequent processing into a common form. For example, human cells, lacking RecG, may over-produce the classic substrate for RecG: a replication fork missing the nascent leading strand which may then be converted, by some as yet uncharacterized mechanism, into a bona fide distinct substrate for the BLM protein. The converse situation is also possible: that in the absence of BLM, the lesion recognized by BLM is converted into a different lesion that can be recognized and repaired by RecG. An additional possibility is that the lesion recognized by BLM and RecG may be the same, yet the two proteins may convert the lesion to different, but still less genomically destabilizing alternative structures. Mechanistic details aside, that the bacterial RecG protein can relieve human genomic instability phenotypes resulting from BLM protein deficiency demonstrates the highly conserved nature of DNA structural lesions and the type of mitigating enzymatic processing required to preserve genomic stability.

The role of BLM in human genomic stabilization is well-established. In contrast, a major mechanistic role of RecG in *E. coli* genomic stabilization has only recently been elucidated. In the absence of RecG, PriA initiates spurious replication forks throughout the bacterial genome, causing poorly-controlled genomic over-replication and compromised viability [46]. In the absence of RecG, cells have an absolute requirement for a ssDNA exonuclease activity, unless PriA is also eliminated [47]. It will be interesting to determine whether human *BLM-* cells share this over-replicated phenotype. Curiously, the *E*. *coli* RecQ protein rather than protecting the cell from aberrant recombination structures as the human BLM and *E. coli* RecG proteins do, seems to act instead to promote their formation [48]. Cells lacking the UvrD-mediated inhibition of recombination and also lacking RecG are killed by formation of intermolecular recombination intermediates (IRIs) that interfere with correct genome segregation to daughter cells. Formation of IRIs is caused by the action of RecQ and partner proteins and causes “death by recombination”. Deletion of RecQ restores cellular viability [49]. An analogous phenotype in human cells might be the elevated formation of anaphase bridges in cells deficient for BLM [50]. One evolutionary interpretation consistent with our functional data here would be that although the ancient common protein ancestor of both RecQ and BLM was preserved in evolutionary descent through both lineages to provide a core helicase domain, the functionality of these proteins diverged in opposite directions. Since there are no RecG homologs by sequence similarity in human cells, it would appear that at least one of the five human RecQ paralogs, BLM, has at least to some extent taken on the important recombination molecular transactions provided to *E. coli* by the RecG protein, and is an example of convergent evolution.

## Conclusion

*E. coli* RecG and human BLM play similar, and to some extent interchangeable, roles in suppressing genomic instability phenotypes when expressed in human cells. Accordingly, given their lack of sequence homology, *E. coli* RecG and human BLM may be an example of convergent evolution.

## Competing interests

The authors declare that they have no competing interests.

## Authors’ contributions

MWK carried out cell line development and molecular analysis, DMS participated in gene cluster instability experiments, WAW subcloned the RecG coding sequence from *E. coli*. AJP directed the studies and wrote the manuscript. All authors read and approved the final manuscript.
